# Predicting educational achievement from DNA

**DOI:** 10.1038/mp.2016.107

**Published:** 2016-07-19

**Authors:** S Selzam, E Krapohl, S von Stumm, P F O'Reilly, K Rimfeld, Y Kovas, P S Dale, J J Lee, R Plomin

**Affiliations:** 1 King's College London, MRC Social, Genetic and Developmental Psychiatry Centre, Institute of Psychiatry, Psychology & Neuroscience, London, UK; 2 Department of Psychology, Goldsmiths University of London, London, UK; 3 Laboratory for Cognitive Investigations and Behavioural Genetics, Tomsk State University, Tomsk, Russia; 4 Department of Speech and Hearing Sciences, University of New Mexico, Albuquerque, NM, USA; 5 Department of Psychology, University of Minnesota Twin Cities, Minneapolis, MN, USA

## Abstract

A genome-wide polygenic score (GPS), derived from a 2013 genome-wide association study (*N*=127,000), explained 2% of the variance in total years of education (*EduYears*). In a follow-up study (*N*=329,000), a new *EduYears* GPS explains up to 4%. Here, we tested the association between this latest *EduYears* GPS and educational achievement scores at ages 7, 12 and 16 in an independent sample of 5825 UK individuals. We found that *EduYears* GPS explained greater amounts of variance in educational achievement over time, up to 9% at age 16, accounting for 15% of the heritable variance. This is the strongest GPS prediction to date for quantitative behavioral traits. Individuals in the highest and lowest GPS septiles differed by a whole school grade at age 16. Furthermore, *EduYears* GPS was associated with general cognitive ability (~3.5%) and family socioeconomic status (~7%). There was no evidence of an interaction between *EduYears* GPS and family socioeconomic status on educational achievement or on general cognitive ability. These results are a harbinger of future widespread use of GPS to predict genetic risk and resilience in the social and behavioral sciences.

## Introduction

Identifying the genetic variants responsible for the ubiquitous heritability of behavioral dimensions and disorders is transforming genetic research in the social and behavioral sciences by making it possible to predict genetic strengths and weaknesses of individuals from DNA alone.^1^ Over the past decade, genome-wide association (GWA) research across the life sciences has revealed that there are almost no genetic variants with large effects on complex traits and common disorders.^2^ This consistent finding implies that the heritability of behavioral traits is due to many genetic variants of small effect. GWA studies of behavioral traits began to be successful as their sample sizes increased sufficiently to detect associations of very small effect size between single-nucleotide polymorphisms (SNPs) and outcome.^3^ Although the largest effect sizes of the associations between SNPs and behavioral traits are very small, it is possible to aggregate the effects of thousands of SNP associations, ranked by effect size, into a SNP genotypic score for a particular trait.^4, 5, 6^ Here, we refer to this SNP genotypic score as a genome-wide polygenic score (GPS).^7^ Although many different labels have been ascribed to polygenic scores that usually include the word *risk*, we prefer GPS. It highlights the genome-wide nature of these polygenic scores and encompasses positive as well as negative effects implied by the normal distribution of polygenic scores.^4^

The largest GWA analysis of a behaviorally relevant trait so far was performed on years of education, which is a proxy for educational achievement and to a lesser extent for learning ability.^8^ Information about the years spent in education is available in many GWA samples because it is a demographic descriptor. In 2013, a GWA analysis of *EduYears* based on 126,559 individuals was published.^9^ The corresponding GPS accounted for 2–3% of the variance in years of education in independent samples.^9, 10^ The latest GWA on years of education published in 2016 included ∼329,000 individuals.^8^ A revised GPS based on this new GWA almost doubled the effect size, with *EduYears* GPS explaining 3.9% of the variance in years of education in an independent sample.^8^

*EduYears* GPS has also been associated with other phenotypes, most notably, measured educational achievement. In a Dutch study, the 2013 *EduYears* GPS accounted for around 2% of the variance in educational achievement in a sample of about 1000 children tested at age 12.^11^ A UK-based longitudinal study of 4500 participants reported significant associations between the 2013 *EduYears* GPS and educational achievement at 7, 11 and 16;^12^ however, the authors did not report the phenotypic variance explained by *EduYears* GPS. In a subsample of the present study of ~3000 individuals, we previously found that the 2013 *EduYears* GPS accounted for about 2% of the variance in educational achievement at age 16.^13^

The present study evaluates the extent to which a GPS constructed on the basis of the published summary statistics of the 2016 GWA analysis of years of education in adulthood predicts educational achievement assessed during the school years, which we have shown to be about 60% heritable estimated by the twin design.^14, 15^ Using effect size estimates from the 2016 *EduYears* GWA analysis, we calculated a GPS for each individual in a sample of 5825 unrelated UK students for whom we had educational achievement scores at ages 7, 12 and 16 based on UK-wide assessments of the national curriculum.

As mentioned, the 2016 *EduYears* GPS is based on a GWA sample almost three times as large as the 2013 GWA (329,000 versus 127,000), and as a result, the amount of variance that *EduYears* GPS accounted for in the discovery sample doubled (~4 versus 2%). Accordingly, here we tested the extent to which the 2016 *EduYears* GPS accounts for more variance in educational achievement than the 2013 *EduYears* GPS. In addition, we addressed two specific questions about the role of *EduYears* GPS for educational achievement.

First, we tested the extent to which the 2016 *EduYears* GPS is associated with general cognitive ability (*g*, aka intelligence) and with family socioeconomic status (SES), both of which phenotypically correlate with educational achievement ~0.40–0.50.^16^ Using summary statistics derived from GWA analyses, a study applying the LD score regression method^17^ identified very high genetic correlations between years of education and childhood IQ (*rg*=0.73).^18^ In a subsample of ~3000 individuals from the current study, the 2013 *EduYears* GPS accounted for ~2% of the variability in *g* at age 16.^19^ We also reported that this GPS explained ~2.5% of the variance in family SES, which refers to the SES of the children's parents.^13^ In the present study, we predicted that the 2016 *EduYears* GPS would yield stronger associations with *g* and family SES than previously found for the 2013 *EduYears* GPS. In addition, we tested whether the 2016 *EduYears* GPS is significantly associated with educational achievement independent of *g* and family SES.

Second, we tested the hypothesis that SES moderates genetic influences on educational achievement and *g,* as predicted by previous studies that observed decreased heritability estimates in low compared with high SES families.^20^ This genotype–environment interaction hypothesis leads to the prediction that *EduYears* GPS is more strongly associated with educational achievement and *g* in high compared with low-SES families. In addition, we tested whether this genotype–environment interaction increased from childhood through adolescence as family SES should have a progressively stronger effect on these aspects of children's lives if the genotype–environment interaction hypothesis is correct.

## Materials and methods

### Participants

This study included unrelated individuals from the multivariate longitudinal Twins Early Development Study that recruited almost 17,000 twin pairs born in England and Wales between 1994 and 1996.^21^ The sample is representative of British families in ethnicity, family SES and parental occupation.^21^ The genotyped subsample is representative of UK census data at first contact (Supplementary Table S1). The Institute of Psychiatry, Psychology and Neuroscience ethics committee (05.Q0706/228) granted project approval and parental consent was obtained prior to data collection.

DNA for 3497 individuals was extracted from saliva samples and hybridized to HumanOmniExpressExome-8v1.2 genotyping arrays at the MRC SGDP Centre Molecular Genetics Laboratories. The raw image data from the array were normalized, pre-processed, and filtered in GenomeStudio according to Illumina Exome Chip SOP v1.4. (http://confluence.brc.iop.kcl.ac.uk:8090/display/PUB/Production+Version&percnt;3A+Illumina+Exome+Chip+SOP+v1.4). In addition, prior to genotype calling, 869 multi-mapping SNPs and 353 samples with call rate <0.95 were removed. The ZCALL program^22^ was used to augment the genotype calling for samples and SNPs that passed the initial QC.

DNA from an additional 3665 samples genotyped earlier in the project was extracted from buccal cheek swabs and genotyped at Affymetrix (Santa Clara, CA, USA). Samples were successfully hybridized to AffymetrixGeneChip 6.0 SNP genotyping arrays (http://www.affymetrix.com/support/technical/datasheets/genomewide_snp6_datasheet.pdf) using experimental protocols recommended by the manufacturer (Affymetrix). The raw image data from the arrays were normalized and pre-processed at the Wellcome Trust Sanger Institute (Hinxton, UK) for genotyping as part of the Wellcome Trust Case Control Consortium 2 (https://www.wtccc.org.uk/ccc2/) according to the manufacturer's guidelines (http://www.affymetrix.com/support/downloads/manuals/genomewidesnp6_manual.pdf). Genotypes for the Affymetrix arrays were called using CHIAMO (https://mathgen.stats.ox.ac.uk/genetics_software/chiamo/chiamo.html).

After initial quality control and genotype calling, the same quality control was performed on the samples genotyped on the Illumina and Affymetrix arrays separately using PLINK,^23^ R^24^ and VCFtools.^25^ Samples were removed from subsequent analyses on the basis of call rate (<0.99), suspected non-European ancestry, heterozygosity, array signal intensity (>4 s.d. from the mean) and relatedness. SNPs were excluded if the minor allele frequency was <0.05%, if more than 1% of genotype data were missing or if the Hardy Weinberg *P*-value was lower than 10^−5^. Non-autosomal markers and indels were removed. Association between the SNP and the array, batch or plate on which samples were genotyped was calculated; SNPs with an effect *P*-value less than 10^−3^ were excluded. A total sample of 5825 samples, with 2698 individuals genotyped on Illumina and 3127 individuals genotyped on Affymetrix, remained after quality control.

Genotypes from the two arrays were separately imputed using the Haplotype Reference Consortium^26^ and Minimac3 1.0.13^27, 28^ available on the *Michigan Imputation Server* (https://imputationserver.sph.umich.edu) as reference data. A series of quality checks were performed before merging data from the two arrays imputation (e.g. array effects, allele frequencies by imputation quality). For the present analyses, we limited our analyses to variants genotyped or imputed at info >0.95 on both arrays, and with Hardy Weinberg Equilibrium test *P*-value >10^−^^5^. After stringent pruning to remove markers in high linkage disequilibrium (*R*^2^>0.1) and excluding high linkage disequilibrium genomic regions so as to ensure that only genome-wide effects were detected, we performed Principal Component Analysis on a subset of 40, 745 autosomal SNPs that remained after applying our quality control criteria, and that overlapped between the two genotyping arrays. To control for population stratification, we regressed the GPS on the first 10 principal components and used the residuals in all subsequent analyses.

### Measures

#### National Curriculum levels age 7 and 12

English and mathematics National Curriculum levels were collected from teachers when the twins were aged 7 (M=7.2, s.d.=0.27) and 12 (M=11.4, s.d.=0.66). National Curriculum data and genotypes were available for 4047 children at age 7 and 2950 at age 12. The assessments are based on a rubric aligned with the UK National Curriculum, which is the standardized core academic curriculum formulated by the National Foundation for Educational Research (NFER) and the Qualifications and Curriculum Authority (QCA) (NFER: http://www.nfer.ac.uk/index.cfm; QCA: http://www.qca.org.uk). After receiving parental consent, teachers were contacted directly via mail. Teacher ratings assessed two main abilities: English (including ‘speaking and listening', ‘reading' and ‘writing') and mathematics (including ‘using and applying mathematics', ‘numbers' and ‘shapes, space and measures').

At age 7 and 12, teachers rated National Curriculum levels on a 5-point and 9-point scale, respectively, with higher scores representing greater ability. Mathematics and English abilities correlated 0.74 and 0.81 at age 7 and 12, respectively. Therefore, we created overall academic achievement mean scores by calculating the standardized mean for the English and mathematics scores for both ages.

#### General Certificate of Secondary Education measures age 16

The General Certificate of Secondary Education (GCSE) is a standardized UK-based examination taken at the end of compulsory education at age 16. In addition to the compulsory core subjects of English, mathematics and science, students can choose from a variety of subjects such as physical education, music, geography, modern foreign languages, and information and communication technology.

GCSE results were obtained by questionnaires sent via mail and by telephone interviews of parents and twins themselves. The grades were coded to range from 4 (G; the minimum pass grade) to 11 (A* the best possible grade). The GCSE score used in this study represents the mean of the compulsory core subjects mathematics and English (if both English language and English literature were taken, a mean grade for English was derived). The two subjects correlated 0.70. We included only mathematics and English grades in the composite score to improve comparability between the educational achievement measures at the different ages. Self-reported GCSE grades of Twins Early Development Study participants show high accuracy, correlating 0.98 English and 0.99 for mathematics grades with data obtained for a subsample from the National Pupil database (NPD: https://www.gov.uk/government/collections/national-pupil-database).^14^ Data for subject grades and genotypes were available for 4301 twins (mean age=16.62, s.d.=0.32).

#### General cognitive ability (*g*)

To measure general cognitive ability, the twins were assessed on various tests including verbal and non-verbal abilities at age 7, 12 and 16. A mean score composite was derived from four tests ('Conceptual Grouping',^29^ ‘Similarities',^30^ ‘Vocabulary',^30^ ‘Picture Completion'^30^) at age 7; three tests (‘Raven's Progressive Matrices',^31^ ‘General Knowledge'^32^ ‘Picture Completion'^30^) at age 12; and two tests ('Raven's Progressive Matrices' and 'Mill Hill Vocabulary test') at age 16. Behavioral and genotypic data were available for 3559 individuals at age 7 (M=7.17, s.d.=0.29); 3349 individuals at age 12 (M=11.46, s.d.=0.64) and 1743 individuals at age 16 (M=16.52, s.d.=0.30). General cognitive ability measures at the different ages correlated on average 0.48. For simplicity we created a general cognitive ability mean composite based on data available at ages 7, 12 and 16. Only participants with data from at least two ages were included (*N*=2228), and mean imputation was performed on those with a missing third measure. We also report results related to general cognitive ability measured at each age individually in Supplementary Table S6.

#### Family SES

A composite of several factors such as parental education and occupation is considered to reflect SES better than any single factor.^33^ Data from 4958 genotyped individuals were available for family SES. This measure represents maternal age at birth of eldest child, the mean score of maternal and paternal highest education level, as well as the respondent's (mother or father) occupation, administered by the Standard Occupational Classification 2000 (Office for National Statistics, 2000) at child age 2, which was the first age of contact.

Small but significant mean differences between girls and boys were found for educational achievement at all ages (Supplementary Table S2). Small age effects were found for educational achievement within each of the three ages (Supplementary Table S2). Therefore, all measures with the exception of SES and *EduYears* GPS were recalculated as standardized residuals corrected for gender and age. To account for a slight negative skew in educational achievement tests at age 7 and 16 and a slight positive skew at age 12, measures were quantile normalized.^34^

### Statistical analyses

#### Genome-wide polygenic scores

We computed GPS for 5825 unrelated individuals using *β*-weights and *P*-values from summary statistics obtained by GWA analysis. Summary statistics were derived from the 2016 GWA study on years of education^8^ with a sample size of 328,918 individuals. It should be noted that the summary statistics we used are slightly different to those of the 2016 *EduYears* study:^8^ here 23andMe data are excluded due to legal restrictions, and an initial release of the UK Biobank data are included (see Supplementary Table S3 for cohort details). GPS based on these modified summary statistics correlated highly (*r*=0.86) with the published GPS^8^ when both GPS were constructed using the Health and Retirement Study as target sample. Quality-controlled SNPs were clumped for linkage disequilibrium in PRSice,^35^ using *R*^2^=0.1 cutoff within a 250-kb window.  In toal, 108,737 SNPs remained after linkage disequilibrium clumping. We used PRSice^35^ to calculate polygenic scores. Firstly, PRSice calculated GPS for each individual in our sample by summing the trait-associated SNPs that are weighted by their effect size derived from GWA analysis. PRSice then performed a regression analysis to test for association between GPS and each of our outcomes (educational achievement at 7, 12, 16, SES and *g*). This is repeated for GPS calculated at a large number of *P*-value thresholds, ranging from 0.001 to 1 (increments of 0.001) in the GWA results, under the high-resolution scoring option in PRSice. Through this high-resolution scoring we identified the ‘best-fit' GPS for all measures (Supplementary Table S4), which were used throughout our analyses for each respective trait. The ‘best-fit' GPS is identified as that which gives the smallest *P*-value for association with outcome among all the regression tests performed on the GPS (see Supplementary Figures S4). Given the multiple testing involved in high-resolution scoring we use an association significance threshold of *P*=0.001, as recommended in Euesden *et al.*^35^

For our GPS analyses, we have more than 80% power to explain 0.2% of the phenotypic variance (see Supplementary Methods S1 for details). To test interactions between different levels of *EduYears* GPS and family SES, we have more than 80% power to detect a small interaction effect of *η*^2^=0.02 (given *α*=0.05; *N*=600; number of groups=4).

We performed regression analyses with *EduYears* GPS as a predictor of educational achievement at ages 7, 12 and 16, as well as of *g* and family SES. To test for potential differences between correlations between *EduYears* GPS and educational achievement at the different ages, we performed Fisher's *r*-to-*z* transformations. We also used multiple regression to test whether associations between *EduYears* GPS and educational achievement remain after controlling for family SES and *g*. We also tested for mean differences in educational achievement between the extreme septiles of *EduYears* GPS at each age using analyses of variance. Finally, interaction effects between *EduYears* GPS and SES on educational achievement and on *g* were analyzed using multiple regression models that included each main effect and the interaction effect term.

## Results

### Polygenic score analyses

**Figure 1:**
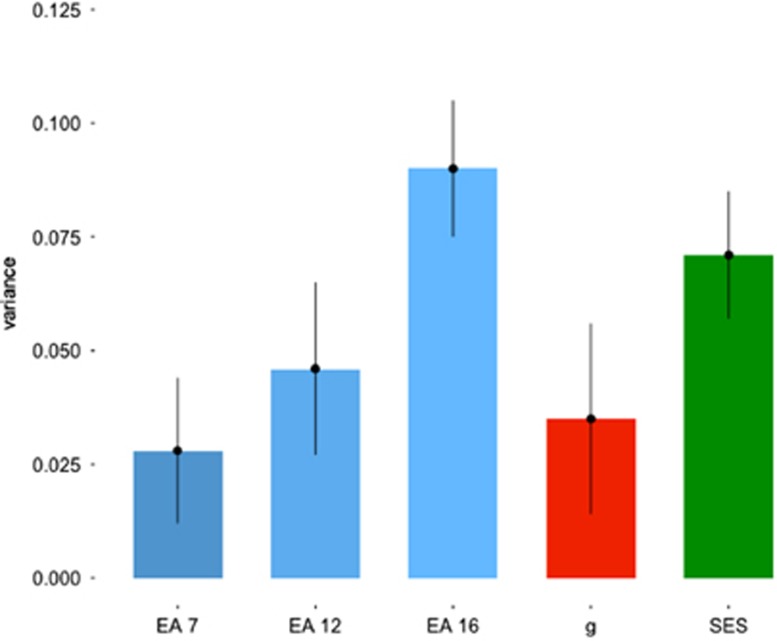
Variance explained (*R*^*2*^) and standard error of *EduYears* GPS predicting: EA 7=educational achievement age 7; EA 12=educational achievement age 12; EA 16=educational achievement age 16; *g*=general cognitive ability; SES=family socioeconomic status; in this analysis and all subsequent analyses, the unique ‘best-fit' GPS was used for each respective trait; see the Materials and methods section for details. GPS, genome-wide polygenic score.

As illustrated in Figure 1, *EduYears* GPS accounted for a significant proportion of variance in educational achievement at all ages, increasing from age 7 (*R*^*2*^=0.028*, P*<0.001) to age 12 (*R*^*2*^=0.046, *P*<0.001) to age 16 (*R*^*2*^=0.091, *P*<0.001). Betas indicated that an increase of one standard deviation in *EduYears* GPS resulted in a *z*-standardized mean educational achievement score increase of 0.17, 0.21 and 0.30 at age 7, 12 and 16, respectively. The increase in association between *EduYears* GPS and educational achievement between age 7 and age 16 was significant, as was the association between age 12 and age 16, but not between age 7 and 12 (Supplementary Table S5).

*EduYears* GPS was also associated with *g* (*R*^*2*^=0.036*, P*<0.001) and family SES (*R*^*2*^=0.073*, P*<0.001) (Figure 1). Additionally, *EduYears* GPS significantly predicted *g* at ages 7, 12 and 16 (Supplementary Table S6); these associations were not statistically different. Because educational achievement, *g*, and family SES are intercorrelated phenotypically (Supplementary Table S6), we tested the effect of *EduYears* GPS on educational achievement independent of *g* and SES by including *g* and SES into a regression model before entering *EduY*ears GPS. After adjusting the *P*-value threshold for multiple testing (see the Materials and methods section), *EduYears* GPS remained a significant predictor of educational achievement at age 16 after accounting for *g* and SES, although the effect size was reduced to 1.2% of the variance explained (Supplementary Table S7).

### Extreme group differences

**Figure 2:**
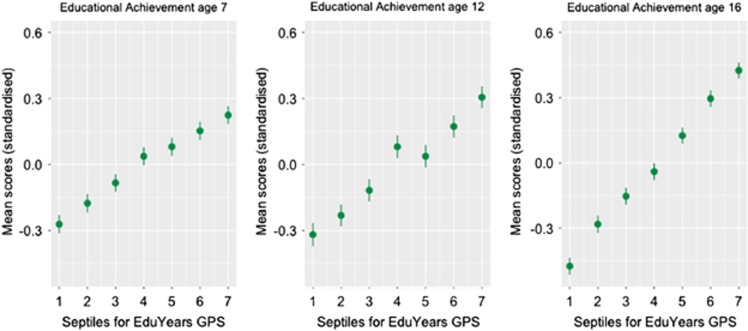
Standardized means and standard errors for educational achievement at age 7, 12 and 16 by genome-wide polygenic score (GPS) septile. *EduYears* GPS was rescored as septiles (1=lowest, 7=highest).

Figure 2 shows the z-standardized mean educational achievement scores by *EduYears* GPS septiles. At all ages, individuals scoring in the highest *EduYears* GPS septile performed on average significantly and substantially better at school than those scoring in the lowest GPS septile (Supplementary Table S8). By age 16, there was almost a standard deviation difference in educational achievement between the lowest and highest GPS groups, which represents a whole school grade difference. Similar results were obtained for *EduYears* GPS extreme quintiles rather than septiles (Supplementary Table S9 and Supplementary Figure S1). Using Monte Carlo integration,^36^ we calculated a substantial non-overlap of 38% between educational achievement distributions at age 16 for the lowest and highest GPS septiles (Supplementary Figure S2).

### Genotype–environment interaction effects

**Figure 3:**
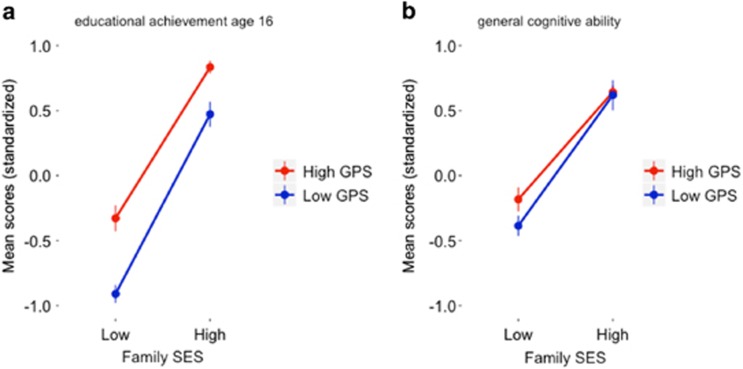
(**a**) Standardized educational achievement mean scores at age 16 by *EduYears* GPS and family SES for individuals scoring in the highest and lowest 20% of the distribution of *EduYears* GPS. There was no evidence for an interaction effect (F(1,605)=1.29, *P*=0.18); (**b**) general cognitive ability mean scores by *EduYears* GPS and family SES for individuals scoring in the highest and lowest 20% of the distribution of *EduYears* GPS. No interaction effect was found (F(1,327)=1.06, *P*=0.30). GPS, genome-wide polygenic score; SES, socioeconomic status.

The genetic influence of *EduYears* GPS on educational achievement at age 16 and on *g* was not greater in high SES than in low-SES families, as would be predicted by the genotype–environment interaction hypothesis described earlier. As illustrated in Figure 3a, at age 16 the difference between low and high GPS groups was similar for low-SES and high-SES groups, despite the higher mean educational achievement of the high-SES group. We also did not find G × E interaction for general cognitive ability (Figure 3b), and educational achievement at ages 7 and 12 (Supplementary Figure S3). Hierarchical multiple regression analyses that tested for G × E interaction using continuous data yielded no significant interactions between *EduYears* GPS and SES as they relate to educational achievement at ages 7, 12 and 16 (Supplementary Table S10) or as they relate to *g* (Supplementary Table S11).

## Discussion

Our results show that DNA can be used to predict educational achievement, especially at the end of the compulsory school years. Although the 2016 *EduYears* GPS accounted for ~4% of the variance in the GWA target trait of years of education in independent samples, we found that the 2016 *EduYears* GPS accounted for 9% of the variance in educational achievement at age 16, tripling the effect size from previous reports^13^ based on the 2013 *EduYears* GPS.^9^ The predictive power of *EduYears* GPS can be seen especially at the extremes of the distribution of GPS scores, suggesting that it is possible to identify individuals early in life at genetic risk and resilience, moving us closer to the possibility of early intervention and personalized learning.^37^

We have previously reported a heritability estimate of 60% for educational achievement at age 16 using a sample from which the present sample was drawn.^14^ The present study demonstrated that *EduYears* GPS predicts 9% of the total variance in educational achievement, thus accounting for only 15% of the heritability estimated by the twin design. However, unlike twin study estimates of heritability, GPS is derived from GWA studies, which are limited to additive effects of the common variants employed on SNP arrays. For this reason, SNP-based estimates of heritability, which have these same limitations, represent the current upper limit for GPS prediction. For educational achievement, SNP-based estimates of heritability are about 30%,^13^ and *EduYears* GPS explains almost one-third of the heritable variance from SNP-based studies at age 16.

We believe that the substantial increase in heritability explained by the 2016 *EduYears* GPS represents a turning point in the social and behavioral sciences because it makes it possible to predict educational achievement for individuals directly from their DNA. Although other variables account for more of the variance of educational achievement, DNA has a unique predictive status in that inherited DNA sequence variation does not change from the single cell with which life begins. For this reason, unlike the case with many other predictors, the correlation between *EduYears* GPS and educational attainment cannot feasibly be interpreted in terms of reverse causation. That is, the correlation between *EduYears* GPS and educational achievement cannot be caused by the effect of educational achievement on inherited DNA sequence variation. In contrast, although *g* predicts much more of the variance of educational achievement at age 16 (29% in our study), this correlation could be confounded by factors related to both educational achievement and *g*, such as social and family risk factors. Similarly, educational achievement at age 7 predicts 35% of the variance of educational achievement at age 16 but this correlation could also be due to other factors, including genetics,^14^ that affect educational achievement at both ages. Moreover, educational achievement and *g* cannot be assessed at earlier stages of development. Family SES, which also predicts substantial variance of educational achievement at age 16 (21% in our study), can be assessed early but this correlation is also likely to be partly caused by other factors, including genetics,^13^ that affect both family SES and educational achievement. Although family SES can be assessed early, it can change over time, whereas DNA variations within individuals are stable across the lifespan. Moreover, family SES is a family-wide index not specific to individual children in a family.

*EduYears* GPS predicts educational achievement independently of *g* and family SES only at age 16, which may be due to the associations between *g,* educational achievement, family SES and *EduYears* GPS. It is possible that family SES and *g* are earlier in the chain of the causal pathway from genetic variants to educational achievement, which may explain the attenuated relationship between *EduYears* GPS and educational achievement at age 7 and 12 after controlling for these variables. Our findings suggest pleiotropic effects of *EduYears* GPS on educational achievement, *g*, and family SES, which are in line with previous reports that describe the genetic overlap between educational achievement, *g,* and family SES.^12, 13, 38^ However, the threefold increase in prediction of educational achievement at age 16 from the 2016 *EduYears* GPS as compared with the 2013 *EduYears* GPS (~3% vs 9%) was not mirrored in the prediction of *g* (~2% vs ~3.5%). The finding that *EduYears* GPS accounts for more variance in educational achievement than in *g* is likely due to the fact that educational achievement is influenced by *g* as well as many other factors that are under genetic influence.^14^

Variance explained by the 2016 *EduYears* GPS in family SES also increased almost threefold compared with previous results with the 2013 EduYears GPS in the a subsample of the current study (~2.5% vs ~7%).^13^ Explaining ~7% in family SES by *EduYears* GPS is impressive for two reasons. First, the children's genotypes are only an approximation of their parents' genotypes; the effect of *EduYears* GPS on SES should be even stronger for the parents' own GPS. Second, our findings account for a third of the SNP-based heritability estimate for family SES (~20%),^39^ which, as noted earlier, represents the upper limit for GWA and GPS studies. With that, our results demonstrate that family SES is genetically influenced and that its genetic effects are also partly shared with educational achievement.

When interpreting the current results, three caveats should be considered. First, the finding that the predictive validity of *EduYears* GPS increases across the school years may be due to increasing approximation of our measures to the *EduYears* GWA target trait of years of education. That is, our measure of educational achievement at age 16 is a standardized examination taken at the end of compulsory education that strongly influences whether pupils go on to higher education. Alternatively, it is also possible that GCSE results are more reliable measures than national curriculum teacher ratings, which might contribute to the difference in variance explained in these variables by *EduYears* GPS. Second, as we measured family SES in a traditional way by including parental education, this could have increased the association of the SES composite with *EduYears* GPS. Although parental education and occupation are related, future studies should investigate if the relationship between *EduYears* GPS and SES varies as a function of different SES indicators. Third, our finding that *EduYears* accounts for 9% of the variance of educational achievement at age 16 needs to be tested for generalization in other samples and beyond the UK.

The finding that individuals' polygenic scores for years of education predict educational achievement entails no necessary policy implications. However, our findings corroborate that individual differences in educational achievement are partly due to DNA differences between children and are not solely created by environmental forces. By creating a dialogue between scientists and policymakers, the introduction of polygenic scores may soon become a useful tool for early prediction and prevention of educational problems and for personalized learning.
