# Erratum: Predicting educational achievement from DNA

**DOI:** 10.1038/mp.2017.203

**Published:** 2017-09-26

**Authors:** S Selzam, E Krapohl, S von Stumm, P F O'Reilly, K Rimfeld, Y Kovas, P S Dale, J J Lee, R Plomin

**Keywords:** Clinical genetics, Paediatric research, Education

**Correction to:** *Molecular Psychiatry* (2017) 22: 267–272; doi: 10.1038/mp.2016.107

Following publication, the authors noticed that some information in the Acknowledgements section was presented incorrectly or omitted. The updated Acknowledgements section follows.

We gratefully acknowledge the ongoing contribution of the participants in the Twins Early Development Study (TEDS) and their families. TEDS is supported by a program grant to RP from the UK Medical Research Council (MR/M021475/1 and previously G0901245), with additional support from the US National Institutes of Health (HD044454; HD059215). SS is supported by the MRC/IoPPN Excellence Award and by the EU Framework Programme 7 (602768). EK and KR are supported by a Medical Research Council studentship. RP is supported by a Medical Research Council Professorship Award (G19/2). The research leading to these results has also received funding from the European Research Council under the European Union's Seventh Framework Programme (FP7/2007-2013)/ERC grant agreement no. 295366.

