# Supplementary material for: Predicting educational achievement from DNA
Source: Mol Psychiatry. 2016 Jul 19;22(2):267–72. doi: 10.1038/mp.2016.107 (PMC5285461; doi:10.1038/mp.2016.107)
Supplement: Supplementary Information [file mp2016107x1.pdf]

Supplementary material

Title

Predicting Educational Achievement from DNA

Authors

Saskia Selzam[1], Eva Krapohl[1], Sophie von Stumm[2], Paul O'Reilly[1], Kaili Rimfeld[1], Yulia Kovas[2,3], Philip S. Dale[4], James J. Lee[5], Robert Plomin*[1]

Supplementary Tables

Table S1. Representativeness of genotyped TEDS sample

	N	Female	A-levels: parental	Mother employed	Father employed
UK census 2001	N/A	50%	32%	49%	89%
TEDS 1st contact	5,825	53%	33%	46%	93%

Note. We used the 2001 UK census data (ONS, 2001; https://www.ons.gov.uk/census/2001censusandearlier/aboutcensus2001) for families with children rather than 2011 UK census data for TEDS twins as they were born between 1994-96.

Table S2. Descriptive Statistics

	N	Mean	SE	Mean F	SE F	Mean M	SE M	skew	min	max	R^2	$R^{2\prime}$
EA 7*	4047	0.05	0.02	0.08	0.02	0.01	0.02	-0.49	-3.96	2.88	<.01	0.02
EA 12*	2950	0.03	0.02	0.07	0.02	-0.02	0.03	0.4	-3.52	4.85	<.01	0.09
EA 16	4301	8.92	0.02	9.01	0.03	8.84	0.03	-0.49	4	11	<.01	<.01
SES*	4958	0.12	0.01	0.14	0.02	0.18	0.02	0.07	-2.6	2.58	N/A	N/A
g comp*	2228	0.11	0.02	0.15	0.03	0.09	0.02	-0.07	-2.71	2.55	nil	nil
g 7*	3559	0.04	0.02	0.05	0.02	0.03	0.03	-0.1	-4	5	nil	<.01
g 12*	3349	0.04	0.02	-0.04	0.02	0.13	0.03	-0.31	-3	3	<.01	0.05
g 16*	1743	0	0.02	-0.03	0.03	0.04	0.04	0.28	-3	3	<.01	<.01

Note. *N* = number of participants; R^2 = variance explained by gender differences; $R^{2\prime}$ = variance explained by age; EA = educational achievement; SES = socioeconomic status; *g* comp = general cognitive ability composite based on *g* measures at age 7, 12 and 16: *g* 7 = general cognitive ability age 7; *g* 12 = general cognitive ability age 12; *g* 16 = general cognitive ability age 16; F = female; M = male; *standardization was required to form composite.

Table S3. Study cohorts included in the *EduYears* GWAS summary statistics

Study	Full name	Sampling	Country	Sample size	Reference	Birth year (Mean/Range)	Fraction female
ACPRC	Manchester Studies of Cognitive Ageing	Population-based	England	1713	1	1923 (1903-1948)	0.71
AGES	Age, Gene/ Environment Susceptibility–Reykjavik Study	Population-based	Iceland	3212	2	1927 (1908-1936)	0.58
ALSPAC	Avon Longitudinal Study of Parents and Children	Population-based birth cohort	England	2877	3	1959 (1948-1963)	1.00
ASPS	Austrian Stroke Prevention Study	Population-based	Austria	777	4,5	1932 (1909-1949)	0.57
BASE-II	Berlin Aging Study II	Population-based	Germany	1619	6	1948 (1925-1983)	0.52
CoLaus	Cohorte Lausannoise	Population-based	Switzerland	3269	7	1950 (1928-1970)	0.53
COPSAC2000	Copenhagen Studies on Asthma in Childhood 2000	Case-control birth cohort	Germany	318	8	1966 (1964-1969)	0.47
CROATIA-Korčula	Croatia Korčula	Population-based (Isolate)	Croatia	842	9	1950 (1909-1977)	0.64
deCODE	deCODE genetics	Population-based	Iceland	46758	10	1945 (1894-1983)	0.57
DHS	Dortmund Health Study	Population-based	Germany	953	11	1949 (1929-1974)	0.53
DIL	Wellcome Trust Diabetes and Inflammation Laboratory	Population-based	England	2578	12	1958 (1958-1958)	0.52
EGCUT1	Estonian Genome Center, University of Tartu	Population-based	Estonia	5597	13	1950 (1905-1980)	0.55
EGCUT2	Same as above	Population-based	Estonia	1328	13	1957 (1911-1979)	0.53
EGCUT3	Same as above	Population-based	Estonia	2047	13	1966 (1930-1982)	0.73
ERF	Erasmus Rucphen Family Study	Family-based	Netherlands	2433	14,15	1952 (1914-1974)	0.55
FamHS	Family Heart Study	Family-based	USA	3483	16,17	1941 (1900-1965)	0.53
FINRISK	The National FINRISK Study	Case-control (Cardiovascular health)	Finland	1685	18	1946 (1923-1977)	0.46
FTC	Finnish Twin Cohort	Family-based	Finland	2418	19	1945 (1910-1972)	0.56
GOYA	Genetics of Overweight Young Adults	Case-control (Obesity)	Denmark	1459	20	1947 (1944-1954)	0.00
GRAPHIC	Genetic Regulation of Arterial Pressure in Humans	Population-based	England	727	21	1951 (1942-1965)	0.53
GS	Generation Scotland	Population-based	Scotland	8776	22	1955 (1909-1981)	0.59
H2000 Cases	Health 2000	Case-control (Metabolic syndrome)	Finland	797	23	1949 (1924-1970)	0.50
H2000 Controls	Same as above	Case-control (Metabolic syndrome)	Finland	819	23	1949 (1924-1969)	0.52
HBCS	Helsinki Birth Cohort Study	Population-based birth cohort	Finland	1617	24	1941 (1934-1944)	0.57
HCS	Hunter Community Study	Population-based	Australia	1946	25	1940 (1920-1951)	0.49
HNRS (CorexB)	Heinz Nixdorf Recall Study	Population-based	Germany	1401	25	1942 (1926-1955)	0.50
HNRS (Oexpr)	Same as above	Same as above	Germany	1347	25	1942 (1926-1955)	0.50
HNRS (Omni1)	Same as above	Same as above	Germany	778	26	1942 (1927-1955)	0.52
HRS	Health and Retirement Study	Population-based	USA	9963	27	1940 (1900-1979)	0.42

Hypergenes	Hypergenes	Case-control	Italy/ UK/ Belgium	815	28	1945 (1914-1971)	0.46
INGI-CARL	Italian Network of Genetic Isolates - Carlantino	Population-based (Isolate)	Italy	947	28	1946 (1910-1975)	0.58
INGI-FVG	Italian Network of Genetic Isolates - Friuli Venezia Giulia	Population-based (Isolate)	Italy	943	29	1951 (1917-1978)	0.60
KORA S3	Kooperative Gesundheitsforschung in der Region Augsburg	Population-based	Germany	2655	29	1945 (1920-1964)	0.51
KORA S4	Same as above	Population-based	Germany	2721	30	1949 (1926-1970)	0.51
LBC1921	Lothian Birth Cohort 1921	Population-based birth cohort	Scotland	515	31	1921 (1921-1921)	0.58
LBC1936	Lothian Birth Cohort 1936	Population-based birth cohort	Scotland	1003	32	1936 (1936-1936)	0.49
LifeLines	The LifeLines Cohort Study	Population-based	Netherlands	12539	33	1960 (1921-1980)	0.58
MCTFR	Minnesota Center for Twin and Family Research	Family-based, but only founders used.	USA	3819	34	1953 (1926-1974)	0.54
MGS	Molecular Genetics of Schizophrenia	Population-based	USA	2313	35,36	1951 (1914-1976)	0.50
MoBa	Mother and Child Cohort of NIPH	Population-based (Nested case-control)	Norway	622	37,38	1971 (1966-1976)	1.00
NBS	Nijmegen Biomedical Study	Population-based	Netherlands	1808	39	1941 (1923-1972)	0.50
NESDA	Netherlands Study of Depression and Anxiety	Case-control (Mental health)	Netherlands	1820	40	1958 (1939-1977)	0.64
NFBC66	Northern Finland Birth Cohort 1966	Population-based	Finland	5297	41,42	1966 (1966-1966)	0.52
NTR	Netherlands Twin Register	Family-based	Netherlands	5246	43	1958 (1917-1989)	0.64
OGP	Ogliastra Genetic Park	Population-based	Italy	370	44	1950 (1916-1976)	0.00
OGP-Talana	Ogliastra Genetic Park-Talana	Population-based (Isolate)	Italy	544	44	1949 (1910-1977)	0.59
ORCADES	Orkney Complex Disease Study	Population-based (Isolate)	Scotland	1828	45	1952 (1914-1979)	0.60
PREVEND	Prevention of Renal and Vascular End-stage Disease	Population-based	Netherlands	3578	46	1948 (1923-1968)	0.48
QIMR	Queensland Institute of Medical Research	Family-based	Australia	8006	47	1956 (1900-1984)	0.59
RS-I	Rotterdam Study Baseline	Population-based	Netherlands	6108	48,49	1922 (1893-1938)	0.60
RS-II	Rotterdam Study Extension of Baseline	Same as above	Netherlands	1667	48,49	1935 (1906-1944)	0.52
RS-III	Rotterdam Study Young	Same as above	Netherlands	3040	48,49	1950 (1910-1960)	0.56
Rush-MAP	Rush University Medical Center - Memory and Aging Project	Community-based	USA	887	50	1921 (1901-1948)	0.72
Rush-ROS	Rush University Medical Center - Religious Orders Study	Community-based	USA	808	51	1921 (1896-1946)	0.66
SardiNIA	SardiNIA Study of Aging	Family-based	Italy	5616	52	1955 (1901-1983)	0.58
SHIP	Study of Health in Pomerania	Population-based	Germany	3556	52	1945 (1918-1971)	0.50
SHIP-TREND	Study of Health in Pomerania	Population-based	Germany	901	53	1956 (1928-1980)	0.57
STR – Salty	Swedish Twin Registry	Family-based	Sweden	4832	52	1951 (1943-1958)	0.52
STR – Twingene	Swedish Twin Registry	Family-based	Sweden	9553	54	1941 (1916-1958)	0.53
THISEAS	The Hellenic Study of Interactions between	Case-control	Greece	829	55	1950 (1909-1979)	0.33

	SNPs & Eating in Atherosclerosis Susceptibility						
TwinsUK	St Thomas' UK Adult Twin Registry	Population-based	England	4012	56	1949 (1919-1978)	1.00
WTCCC58C	1958 British Birth Cohort	Population-based	England	2804	57	1958 (1958-1958)	0.48
YFS	The Cardiovascular Risk in Young Finns Study	Population-based	Finland	2029	58	1969 (1962-1977)	0.55
UKB	UK Biobank	Population-based	UK	111349	59	1951 (1934-1970)	0.53

Note. Adapted from Okbay et al., *Nature*, in press[60]

Table S4. Numbers of SNPs per threshold used in creating *EduYears* GPS

pT	Number of SNPs
0.001	2,162
0.01	7,303
0.05	19,415
0.055[1]	20,582
0.063[2,3]	22,384
0.067[4,5]	23,316
0.072[6]	24,377
0.1	30,086
0.2	46,636
0.3	60,012
0.362[7]	67,265
0.364[8]	67,470
0.4	71,382
0.5	81,149

Note. pT = *P*-value threshold; [1]'Best-fit' GPS for *g* 16; [2]'Best-fit' GPS for *g* 7; [3]'Best-fit' GPS for *g* composite; [4]'Best-fit' GPS for *g* 12; [5]'Best-fit' GPS for EA 16; [6]'Best-fit' GPS for family SES; [7]'Best-fit' GPS for EA 12; [8]'Best-fit' GPS for EA 7.

Table S5. Testing significance of differences of the correlations between *EduYears* GPS and educational achievement at ages 7, 12 and 16.

	z	*P*-value
*r*GPS-EA7 - *r*GPS-EA12	1.65	0.10
*r*GPS-EA12 - *r*GPS-EA16	3.88	< 0.001
*r*GPS-EA7 - *r*GPS-EA16	6.29	< 0.001

Note. GPS = *EduYears* GPS; EA 7 = educational achievement age 7; EA 12 = educational achievement age 12; EA 16 = educational achievement age 16; *r* = Pearson's correlation.

Table S6. Correlations among all variables

	EA 7	EA 12	EA 16	*g* comp	*g* 7	*g* 12	*g* 16	SES	GPS
EA7	1								
EA12	0.60***	1							
EA16	0.59***	0.66***	1						
g	0.50***	0.53***	0.61***	1					
g 7	0.45***	0.41***	0.46***	0.76***	1				
g 12	0.44***	0.48***	0.53***	0.83***	0.45***	1			
g 16	0.41***	0.48***	0.58***	0.83***	0.42***	0.59***	1		
SES	0.33***	0.35***	0.46***	0.37***	0.33***	0.34***	0.33***	1	
GPS	0.17***	0.21***	0.30***	0.19***	0.15***	0.19***	0.20***	0.27***	1

Note. EA 7 = educational achievement age 7; EA 12 = educational achievement age 12; EA 16 = educational achievement age 16; *g* comp = general cognitive ability composite; *g* 7 = general cognitive ability age 7; *g* 12 = general cognitive ability age 12; *g* 16 = general cognitive ability age 16; SES = family socioeconomic status; GPS = genome-wide polygenic score; ***P < 0.001; *unique ‘best-fit’ GPS was used for each respective trait, see methods section for details.

Table S7. Predicting educational achievement at age 7, 12 and 16 from *g, SES* and *EduYears* GPS: Multiple Regression Analysis

	Dependent variable:		
	EA 7	EA 12	EA 16
g	**0.44*****	**0.46*****	**0.50*****
	(0.40 - 0.48)	(0.38 - 0.48)	(0.45 - 0.52)
SES	**0.14*****	**0.17****	**0.25****
	(0.09 - 0.19)	(0.11 - 0.22)	(0.22 - 0.30)
GPS	0.03	0.07*	**0.11*****
	(-0.01 - 0.07)	(-0.01 - 0.11)	(0.07 - 0.14)
N	1,738	1,035	1,763
R^2	0.27	0.32	0.45
Adjusted R^2	0.27	0.31	0.45
Residual Std. Error	0.85 (df = 1,734)	0.80 (df = 1,031)	0.72 (df = 1,759)
F Statistic	212.50*** (df = 3; 1,734)	158.81*** (df = 3; 1,031)	474.64*** (df = 3; 1,759)

Note. Standardized coefficients are presented; 95% Confidence Intervals in brackets; **P* < 0.05; ***P* < 0.01; ****P* < 0.001; unique 'best-fit' GPS was used for each respective trait.

Table S8. Testing septile extreme group differences at age 7, 12 and 16 for *EduYears* GPS: Analysis of Variance

	N	*lower extreme Mean (SE)*	*upper extreme Mean (SE)*	*F*	*d*
EA 7	1,139	-0.26(0.04)	0.25(0.04)	**72.84*****	0.51
EA 12	754	-0.32(0.05)	0.31(0.05)	**74.46*****	0.63
EA 16	1,127	-0.47(0.04)	0.43(0.04)	**284.88*****	0.90

Note. Lower and upper extreme represent lowest and highest GPS septiles; *d* = Cohen's *d*; ****P* < 0.001; unique 'best-fit' GPS was used for each respective trait.

Table S9. Testing quintile extreme group differences at age 7, 12 and 16 for *EduYears* GPS: Analysis of Variance

	N	*lower extreme Mean (SE)*	*upper extreme Mean (SE)*	*F*	*d*
EA 7	1,585	-0.24(0.03)	0.21(0.03)	**82.01*****	0.45
EA 12	1,054	-0.28(0.04)	0.26(0.04)	**80.31*****	0.54
EA 16	1,703	-0.41(0.03)	0.40(0.03)	**319.26*****	0.81

Note. Lower and upper extreme represent lowest and highest GPS quintiles; *d* = Cohen's *d*; ****P* < 0.001; unique 'best-fit' GPS was used for each respective trait.

Table S10. Testing for GPSxSES interaction at age 7, 12 and 16: Multiple Regression Analysis

	Dependent variable:		
	EA 7	EA 12	EA 16
GPS	**0.09***	**0.13***	**0.19***
	(0.05 - 0.12)	(0.09 - 0.17)	(0.16 - 0.22)
SES	**0.31***	**0.31***	**0.41***
	(0.28 - 0.35)	(0.27 - 0.35)	(0.40 - 0.46)
GPS*SES	-0.01	0.02	-0.02
	(-0.04 - 0.02)	(-0.01 - 0.06)	(-0.05 - 0.01)
N	3,848	2,341	3,804
R^2	0.12	0.14	0.25
Adjusted R^2	0.12	0.14	0.25
Residual Std. Error	0.94 (df = 3,844)	0.92 (df = 2,337)	0.86 (df = 3,800)
F Statistic	168.85*** (df = 3; 3,844)	124.23*** (df = 3; 2,337)	415.80*** (df = 3; 3,800)

Note. Standardized coefficients are presented; 95% Confidence Intervals in brackets; **P* < 0.05; ***P* < 0.01; ****P* < 0.001; unique 'best-fit' GPS was used for each respective trait.

Table S11. Testing GPSxSES interaction for general cognitive ability: Multiple Regression Analysis

	Dependent variable:
	g
GPS	**0.11***
	(0.07 - 0.16)
SES	**0.37***
	(0.32 - 0.41)
GPS*SES	-0.03
	(-0.07 - 0.01)
N	1,956
R^2	0.15
Adjusted R^2	0.15
Residual Std. Error	0.92 (df = 1,952)
F Statistic	118.32*** (df = 3; 1,952)

Note. Standardized coefficients are presented; 95% Confidence Intervals in brackets; **P* < 0.05; ***P* < 0.01; ****P* < 0.001; unique 'best-fit' GPS for *g* was used.

Supplementary Figures

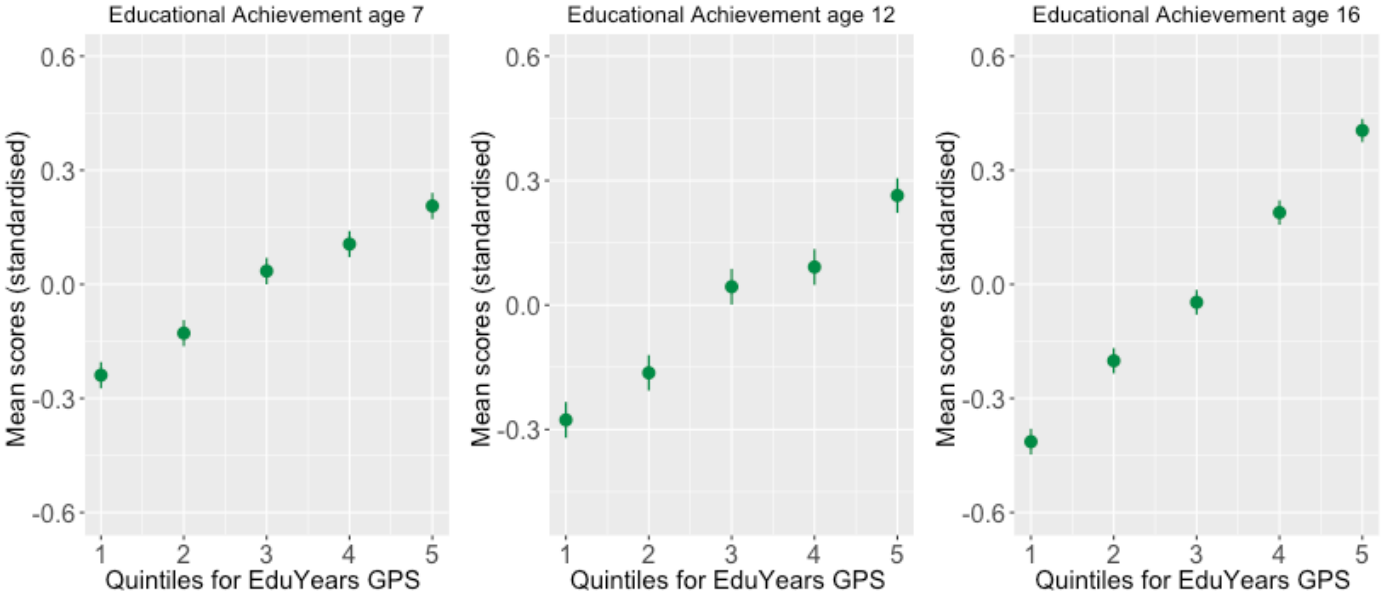

Figure S1. Standardized means and standard errors for educational achievement at age 7, 12 and 16 by genome-wide polygenic score (GPS) quintile. *EduYears* GPS were rescored as quintiles (1=lowest, 5=highest); unique 'best-fit' GPS was used for each respective trait.

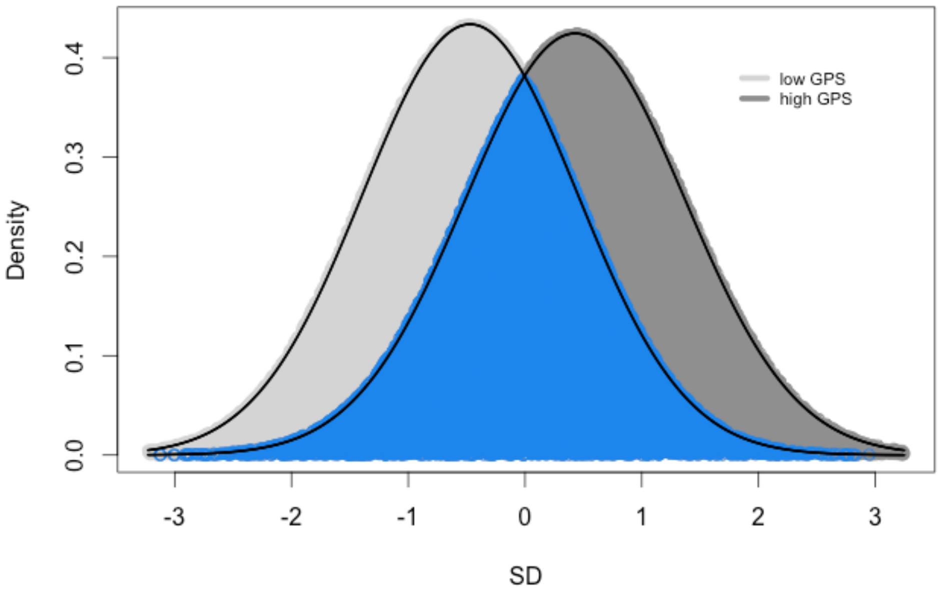

Figure S2. SD = standard deviation; Probability density functions of standardized educational achievement scores age 16 for the lowest *EduYears* GPS septile (low GPS) and highest *EduYears* GPS septile (high GPS). The area in blue indicates the overlap between the distributions.

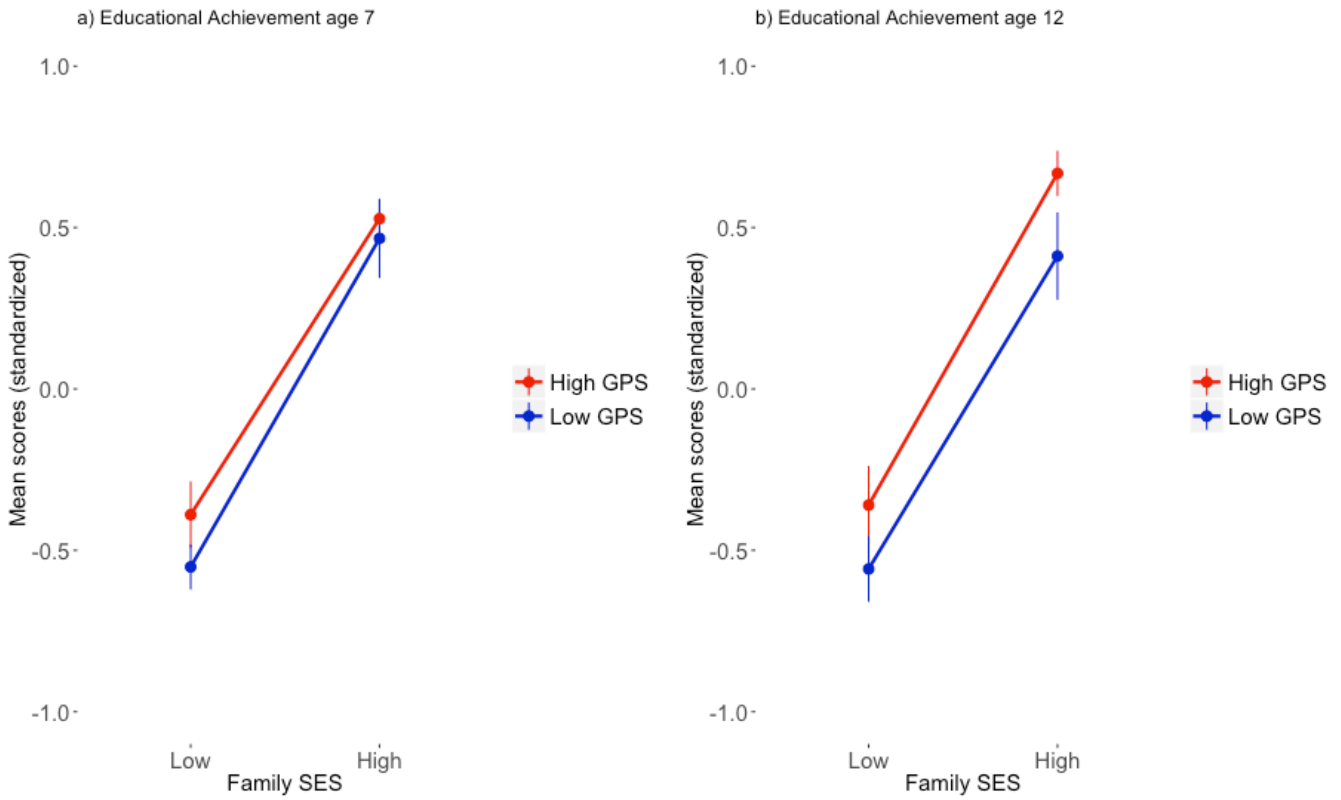

Figure S3. Testing for GxE interaction: Standardized educational achievement mean scores and error bars at a) age 7 and b) age 12 by *EduYears* GPS and family SES for individuals scoring in the highest and lowest quintiles of the distribution for both variables. No interaction effect was found at either age 7 ($F(1,617) = 0.29$, $P = 0.59$) or age 12 ($F(1,362) = 0.06$, $P = 0.80$); unique 'best-fit' GPS was used for both traits.

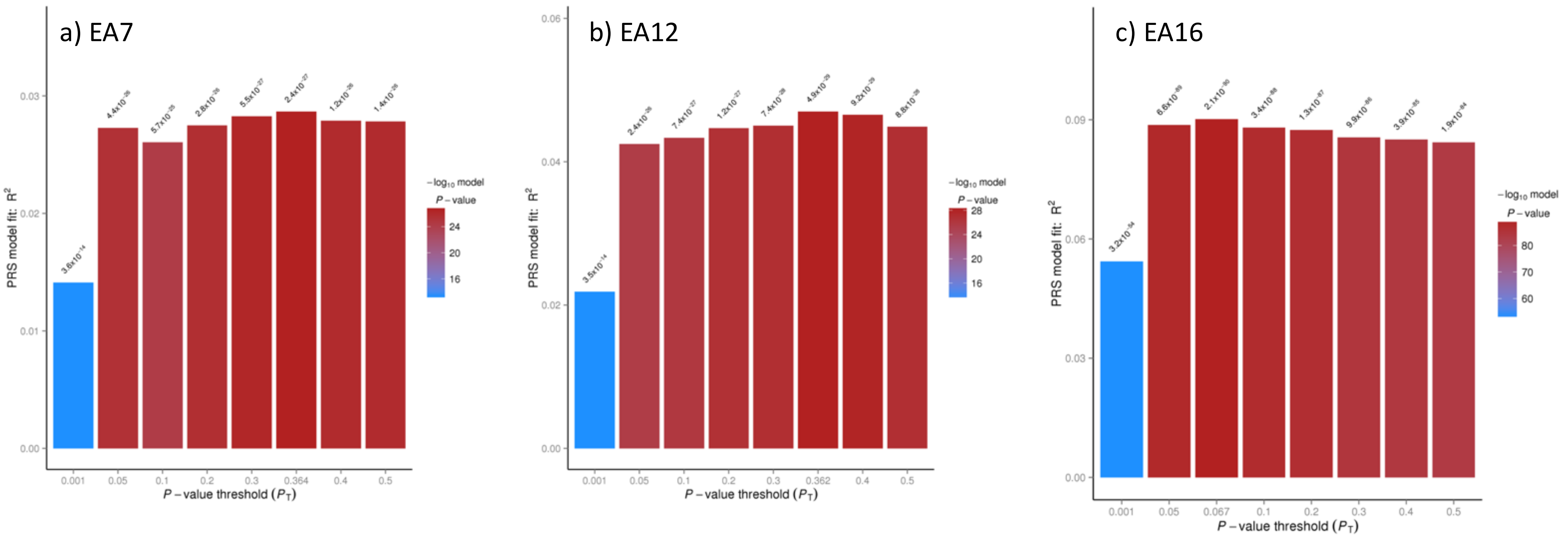

Figure S4. *EduYears* Genome-wide polygenic scores (GPS) explaining variance (R^2) in educational achievement at a) age 7, b) age 12 and c) age 16. Different significance thresholds were used to include SNPs related to years of education ranging from 0.001 to 0.50. Using high-resolution scoring implemented in the PRSice software[61], a series of regression analyses determined the most predictive threshold to compute a GPS for educational achievement at all ages. The uncorrected P-values adjacent to each bar represent the statistical significance of the association between *EduYear*s GPS and the respective trait; see Supplementary Table S10 for the ‘best-fit’ GPS *P*-value threshold for educational achievement at age 7, 12 and 16.

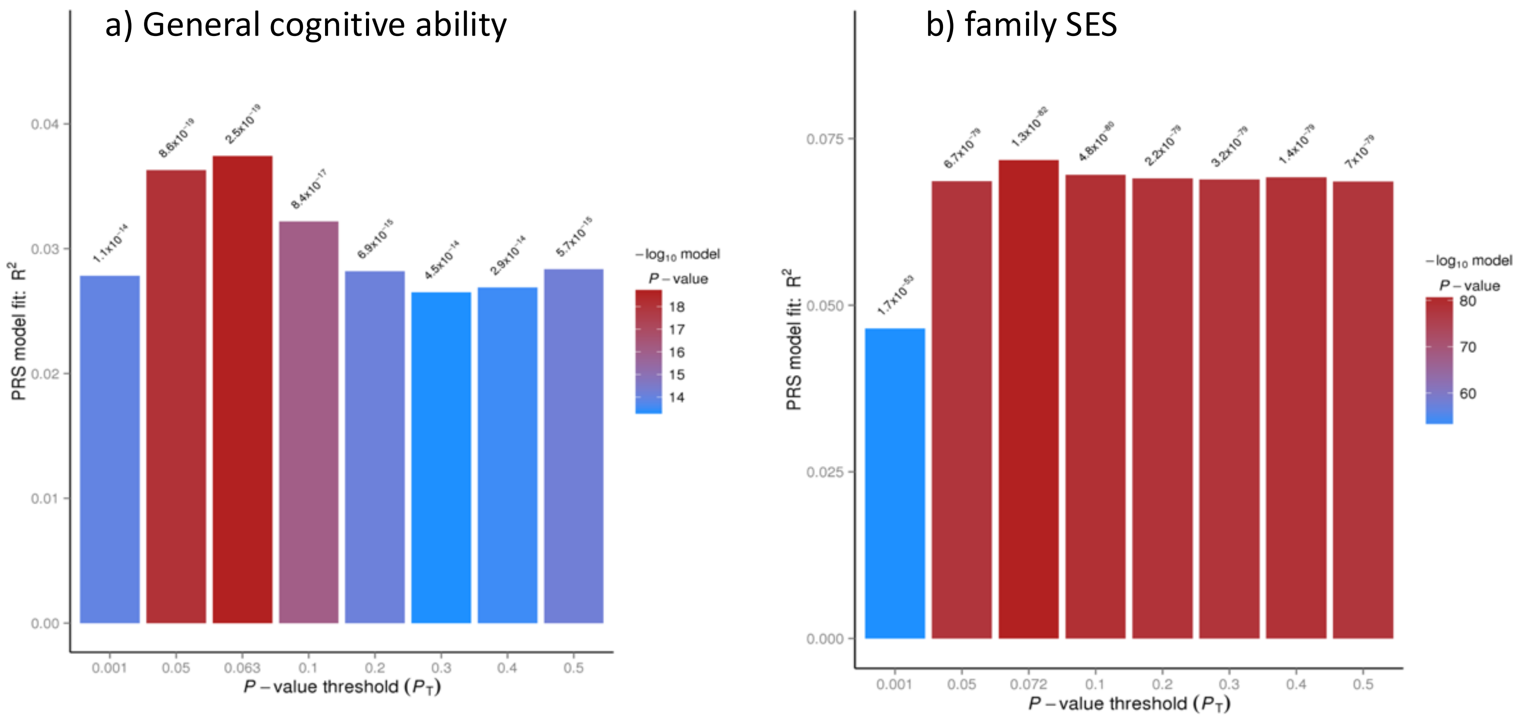

Figure S5. *EduYears* Genome-wide polygenic scores (GPS) explaining variance (R^2) in a) general cognitive ability and b) family SES. Different significance thresholds were used to include SNPs related to years of education ranging from 0.001 to 0.50. Using high-resolution scoring implemented in the PRSice software[61], a series of regression analyses determined the most predictive threshold to compute a GPS for educational achievement at all ages. The uncorrected P-values adjacent to each bar represent the statistical significance of the association between *EduYears* GPS and the respective trait; see Supplementary Table S10 for the 'best-fit' GPS *P*-value threshold for general cognitive ability and family SES.

Supplementary Methods

Methods S1. According to the AVENGEME software[62], a GPS constructed on the basis of a GWA discovery sample size of 328,918 in our target sample including 4,301 individuals (based on the sample size of the educational achievement measure at age 16) has more than 80% power to explain 0.2% of the phenotypic variance under the following circumstances; number of independent SNPs in the GPS = 50,000; proportion of total variance explained by genetic effects in discovery sample = 4%; covariance between genetic effect sizes in the discovery and target sample = 2%; proportion of SNPs with no effects on discovery trait = 99%; Range of P-values from GWA study summary statistics = 0 – 1.

References

1. Rabbitt, P. M. A. *et al.* The University of Manchester Longitudinal Study of Cognition in Normal Healthy Old Age, 1983 through 2003. *Aging Neuropsychol. Cogn.* **11,** 245–279 (2004).
2. Harris, T. B. *et al.* Age, Gene/Environment Susceptibility-Reykjavik Study: multidisciplinary applied phenomics. *Am. J. Epidemiol.* **165,** 1076–1087 (2007).
3. Fraser, A. *et al.* Cohort Profile: the Avon Longitudinal Study of Parents and Children: ALSPAC mothers cohort. *Int. J. Epidemiol.* **42,** 97–110 (2013).
4. Schmidt, R. *et al.* Assessment of cerebrovascular risk profiles in healthy persons: definition of research goals and the Austrian Stroke Prevention Study (ASPS). *Neuroepidemiology* **13,** 308–313 (1994).
5. Schmidt, R., Fazekas, F., Kapeller, P., Schmidt, H. & Hartung, H. P. MRI white matter hyperintensities: three-year follow-up of the Austrian Stroke Prevention Study. *Neurology* **53,** 132–139 (1999).
6. Bertram, L. *et al.* Cohort profile: The Berlin Aging Study II (BASE-II). *Int. J. Epidemiol.* **43,** 703–712 (2014).
7. Firmann, M. *et al.* The CoLaus study: a population-based study to investigate the epidemiology and genetic determinants of cardiovascular risk factors and metabolic syndrome. *BMC Cardiovasc. Disord.* **8,** 1 (2008).
8. Bisgaard, H. The Copenhagen Prospective Study on Asthma in Childhood (COPSAC): design, rationale, and baseline data from a longitudinal birth cohort study. *Ann. Allergy Asthma Immunol. Off. Publ. Am. Coll. Allergy Asthma Immunol.* **93,** 381–389 (2004).
9. Rudan, I. *et al.* '10 001 Dalmatians:' Croatia Launches Its National Biobank. *Croat. Med. J.* **50,** 4–6 (2009).
10. Styrkarsdottir, U. *et al.* Nonsense mutation in the LGR4 gene is associated with several human diseases and other traits. *Nature* **497,** 517–520 (2013).

11. Pfaffenrath, V. *et al.* Regional variations in the prevalence of migraine and tension-type headache applying the new IHS criteria: the German DMKG Headache Study. *Cephalalgia Int. J. Headache* **29,** 48–57 (2009).
12. Strachan, D. P. *et al.* Lifecourse influences on health among British adults: effects of region of residence in childhood and adulthood. *Int. J. Epidemiol.* **36,** 522–531 (2007).
13. Nelis, M. *et al.* Genetic Structure of Europeans: A View from the North–East. *PLOS ONE* **4,** e5472 (2009).
14. Sayed-Tabatabaei, F. A. *et al.* Heritability of the function and structure of the arterial wall: findings of the Erasmus Rucphen Family (ERF) study. *Stroke J. Cereb. Circ.* **36,** 2351–2356 (2005).
15. Sleegers, K. *et al.* Cerebrovascular risk factors do not contribute to genetic variance of cognitive function: the ERF study. *Neurobiol. Aging* **28,** 735–741 (2007).
16. Higgins, M. *et al.* NHLBI Family Heart Study: objectives and design. *Am. J. Epidemiol.* **143,** 1219–1228 (1996).
17. O'Donnell, C. J. *et al.* Genome-wide association study for coronary artery calcification with follow-up in myocardial infarction. *Circulation* **124,** 2855–2864 (2011).
18. Vartiainen, E. *et al.* Thirty-five-year trends in cardiovascular risk factors in Finland. *Int. J. Epidemiol.* **39,** 504–518 (2010).
19. Kaprio, J. The Finnish Twin Cohort Study: An Update. *Twin Res. Hum. Genet.* **16,** 157–162 (2013).
20. Paternoster, L. *et al.* Genome-Wide Population-Based Association Study of Extremely Overweight Young Adults – The GOYA Study. *PLOS ONE* **6,** e24303 (2011).
21. Tobin, M. D. *et al.* Common variants in genes underlying monogenic hypertension and hypotension and blood pressure in the general population. *Hypertension* **51,** 1658–1664 (2008).
22. Smith, B. H. *et al.* Cohort Profile: Generation Scotland: Scottish Family Health Study (GS:SFHS). The study, its participants and their potential for genetic research on health and illness. *Int. J. Epidemiol.* **42,** 689–700 (2013).

23. Aromaa, A. *Health and functional capacity in Finland : Baseline results of the Health 2000 health examination survey*. (National Public Health Institute = Kansanterveyslaitos, 2004).
24. Barker, D. J. P., Osmond, C., Forsén, T. J., Kajantie, E. & Eriksson, J. G. Trajectories of growth among children who have coronary events as adults. *N. Engl. J. Med.* **353,** 1802–1809 (2005).
25. McEvoy, M. *et al.* Cohort profile: The Hunter Community Study. *Int. J. Epidemiol.* **39,** 1452–1463 (2010).
26. Mahabadi, A. A. *et al.* The Heinz Nixdorf Recall study and its potential impact on the adoption of atherosclerosis imaging in European primary prevention guidelines. *Curr. Atheroscler. Rep.* **13,** 367–372 (2011).
27. Sonnega, A. *et al.* Cohort Profile: the Health and Retirement Study (HRS). *Int. J. Epidemiol.* **43,** 576–585 (2014).
28. Salvi, E. *et al.* Genomewide association study using a high-density single nucleotide polymorphism array and case-control design identifies a novel essential hypertension susceptibility locus in the promoter region of endothelial NO synthase. *Hypertension* **59,** 248–255 (2012).
29. Esko, T. *et al.* Genetic characterization of northeastern Italian population isolates in the context of broader European genetic diversity. *Eur. J. Hum. Genet. EJHG* **21,** 659–665 (2013).
30. Wichmann, H.-E., Gieger, C., Illig, T. & MONICA/KORA Study Group. KORA-gen--resource for population genetics, controls and a broad spectrum of disease phenotypes. *Gesundheitswesen Bundesverb. Ärzte Öffentl. Gesundheitsdienstes Ger.* **67 Suppl 1,** S26–30 (2005).
31. Deary, I. J., Whiteman, M. C., Starr, J. M., Whalley, L. J. & Fox, H. C. The impact of childhood intelligence on later life: following up the Scottish mental surveys of 1932 and 1947. *J. Pers. Soc. Psychol.* **86,** 130–147 (2004).
32. Deary, I. J. *et al.* The Lothian Birth Cohort 1936: a study to examine influences on cognitive ageing from age 11 to age 70 and beyond. *BMC Geriatr.* **7,** 28 (2007).
33. Stolk, R. P. *et al.* Universal risk factors for multifactorial diseases: LifeLines: a three-generation population-based study. *Eur. J. Epidemiol.* **23,** 67–74 (2008).

34. Miller, M. B. *et al.* The Minnesota Center for Twin and Family Research genome-wide association study. *Twin Res. Hum. Genet. Off. J. Int. Soc. Twin Stud.* **15,** 767–774 (2012).

35. Sanders, A. R. *et al.* No significant association of 14 candidate genes with schizophrenia in a large European ancestry sample: implications for psychiatric genetics. *Am. J. Psychiatry* **165,** 497–506 (2008).

36. Shi, J. *et al.* Common variants on chromosome 6p22.1 are associated with schizophrenia. *Nature* **460,** 753–757 (2009).

37. Magnus, P. *et al.* Cohort profile: the Norwegian Mother and Child Cohort Study (MoBa). *Int. J. Epidemiol.* **35,** 1146–1150 (2006).

38. Irgens, L. M. The Medical Birth Registry of Norway. Epidemiological research and surveillance throughout 30 years. *Acta Obstet. Gynecol. Scand.* **79,** 435–439 (2000).

39. Wetzels, J. F. M., Kiemeney, L. a. L. M., Swinkels, D. W., Willems, H. L. & den Heijer, M. Age- and gender-specific reference values of estimated GFR in Caucasians: the Nijmegen Biomedical Study. *Kidney Int.* **72,** 632–637 (2007).

40. Penninx, B. W. J. H. *et al.* The Netherlands Study of Depression and Anxiety (NESDA): rationale, objectives and methods. *Int. J. Methods Psychiatr. Res.* **17,** 121–140 (2008).

41. Sabatti, C. *et al.* Genome-wide association analysis of metabolic traits in a birth cohort from a founder population. *Nat. Genet.* **41,** 35–46 (2009).

42. Rantakallio, P. Groups at risk in low birth weight infants and perinatal mortality. *Acta Paediatr. Scand.* **193,** Suppl 193:1+ (1969).

43. Boomsma, D. I. *et al.* Netherlands Twin Register: from twins to twin families. *Twin Res. Hum. Genet. Off. J. Int. Soc. Twin Stud.* **9,** 849–857 (2006).

44. Pistis, G. *et al.* High Differentiation among Eight Villages in a Secluded Area of Sardinia Revealed by Genome-Wide High Density SNPs Analysis. *PLOS ONE* **4,** e4654 (2009).

45. McQuillan, R. *et al.* Runs of homozygosity in European populations. *Am. J. Hum. Genet.* **83,** 359–372 (2008).

46. Hillege, H. L. *et al.* Urinary albumin excretion predicts cardiovascular and noncardiovascular mortality in general population. *Circulation* **106,** 1777–1782 (2002).
47. Martin, N. W. *et al.* Educational Attainment: A Genome Wide Association Study in 9538 Australians. *PLOS ONE* **6,** e20128 (2011).
48. Hofman, A. *et al.* The Rotterdam Study: 2012 objectives and design update. *Eur. J. Epidemiol.* **26,** 657–686 (2011).
49. Estrada, K. *et al.* GRIMP: a web- and grid-based tool for high-speed analysis of large-scale genome-wide association using imputed data. *Bioinforma. Oxf. Engl.* **25,** 2750–2752 (2009).
50. Bennett, D. A. *et al.* Overview and findings from the rush Memory and Aging Project. *Curr. Alzheimer Res.* **9,** 646–663 (2012).
51. Bennett, D. A., Schneider, J. A., Arvanitakis, Z. & Wilson, R. S. Overview and findings from the religious orders study. *Curr. Alzheimer Res.* **9,** 628–645 (2012).
52. Pilia, G. *et al.* Heritability of Cardiovascular and Personality Traits in 6,148 Sardinians. *PLOS Genet* **2,** e132 (2006).
53. Völzke, H. *et al.* Cohort profile: the study of health in Pomerania. *Int. J. Epidemiol.* **40,** 294–307 (2011).
54. Magnusson, P. K. E. *et al.* The Swedish Twin Registry: establishment of a biobank and other recent developments. *Twin Res. Hum. Genet. Off. J. Int. Soc. Twin Stud.* **16,** 317–329 (2013).
55. Theodoraki, E. V. *et al.* Fibrinogen beta variants confer protection against coronary artery disease in a Greek case-control study. *BMC Med. Genet.* **11,** 28 (2010).
56. Moayyeri, A., Hammond, C. J., Valdes, A. M. & Spector, T. D. Cohort Profile: TwinsUK and Healthy Ageing Twin Study. *Int. J. Epidemiol.* **42,** 76–85 (2013).
57. Power, C. & Elliott, J. Cohort profile: 1958 British birth cohort (National Child Development Study). *Int. J. Epidemiol.* **35,** 34–41 (2006).
58. Raitakari, O. T. *et al.* Cohort profile: the cardiovascular risk in Young Finns Study. *Int. J. Epidemiol.* **37,** 1220–1226 (2008).

59. Sudlow, C. *et al.* UK Biobank: An Open Access Resource for Identifying the Causes of a Wide Range of Complex Diseases of Middle and Old Age. *PLOS Med* **12,** e1001779 (2015).
60. Okbay, A. *et al.* Genome-wide association study identifies 74 loci associated with educational attainment. *Nature* (in press).
61. Euesden, J., Lewis, C. M. & O'Reilly, P. F. PRSice: Polygenic Risk Score software. *Bioinformatics* **31,** 1466–1468 (2015).
62. Palla, L. & Dudbridge, F. A Fast Method that Uses Polygenic Scores to Estimate the Variance Explained by Genome-wide Marker Panels and the Proportion of Variants Affecting a Trait. Am. J. Hum. Genet. 97, 250–259 (2015).
